# Macrofluidic recirculating model of skeletal metastasis

**DOI:** 10.1038/s41598-019-50577-3

**Published:** 2019-10-18

**Authors:** Takahiro Osawa, Wenchu Wang, Jinlu Dai, Evan T. Keller

**Affiliations:** 1 Department of Urology, University of Michigan, Michigan, USA; 2 Biointerfaces Institute, University of Michigan, Michigan, USA

**Keywords:** Cancer microenvironment, Bone metastases

## Abstract

While microfluidic systems model aspects of metastasis, they are limited to artificially created tissues of limited complexity. We set out to develop an *in vitro* model of tumor cell migration from a primary tumor to a distant site that allows use of tissue. Accordingly, we created a macrofluidic model using culture plate wells connected with type I collagen-coated large bore tubing and has recirculating media. Green fluorescent protein-positive prostate carcinoma cells in a hydrogel or excised tumor xenografts from mice were placed into primary tumor sites and either human bone stromal cells (HS-5) in a hydrogel or human-derived bone chips were seeded into metastatic sites. Cells from the primary sites migrated to and grew in metastatic sites. Bone enhanced growth at metastatic sites and established a CXCL12 gradient that was higher in metastatic versus primary sites. AMD3100-mediated inhibition of CXCL12 function reduced the number of cells targeting the bone at the metastatic sites. In summary, we have developed a macrofluidic metastasis model that allows incorporation of tumor and metastatic microenvironment tissues and models chemotaxis. This system allows for incorporation of tumor heterogeneity and inclusion of an intact microenvironment. These features will facilitate identification of mechanisms and therapeutics for bone metastasis.

## Introduction

More than 80% of men with advanced prostate cancer develop bone metastases^1^ and autopsy studies of men who died of prostate cancer revealed bone metastases in nearly 90% of the patients examined^2^. Skeletal metastases result in skeletal-related events that cause pain, therefore they greatly impact the patients quality of life (QOL)^3,4^. Thus, it is important that we continue to identify mechanisms and key therapeutic targets that promote bone metastasis to both improve survival and enhance QOL^5^.

Traditional approaches used in cancer research involve *in vitro* culturing tumor cells on 2D surfaces and the use of *in vivo* models, which poorly correlate with human disease states. *In vitro* 2D cell cultures oversimplify the biological environment of a tumor, which is influenced by intrinsic molecular cascades and external keys from its surrounding microenvironment^6^. Classical assays (e.g. Boyden chamber) have been widely used to research cell migration in response to chemotactic gradients, particularly cancer cell invasion and migration. However, they do not provide tight control over the local environment, complex interactions cannot be accurately analyzed, and imaging is challenging^7–9^.

Unlike cancer cells cultured in 2D, those cultured in 3D reveal a rounded shape, forming clusters that are more typical of tumors *in vivo*^10,11^. Cancer cells grown in 2D versus 3D also show differential gene expression profiles for key genes involved in angiogenesis, cell migration, and invasion^12–14^. Although murine *in vivo* models have been developed to research the extravasation process and bone metastasis^15–17^, they do not replicate human-specific characteristics relating to tumors, stem cell differentiation, and their responses to therapeutic drugs. This is because the physiology, tumor cell interactions with the innate immune system, metastasis, and the cells themselves are different from those in humans^18,19^.

In an attempt to incorporate a 3D approach that can be used to evaluate multiple tissues, many investigators have developed microfluidic models. These models provide useful systems to investigate complex phenomena using controlled biochemical and biophysical microenvironments and allow for high resolution real time imaging^20^. In addition, several microfluidic models have been developed to investigate mechanisms of invasion including intravasation^21^ and extravasation^22^. Furthermore, an *in vitro* 3D microfluidic model of the tumor vascular interface was designed to integrate live imaging, precise control of microenvironmental factors, and endothelial barrier measurement^21^. While these microfluidic models have several advantages for research of tumor biology, they are only capable of evaluating small cell numbers in artificially constructed microenvironments.

In an approach to complement microfluidic models, we develop here a novel *in vitro* model that is capable of using *ex vivo* tumor tissue and tissued derived from the microenvironment in a recirculating system to recapitulate the *in vivo* microenvironment to investigate prostate carcinoma metastasis. In this model, we use 6-well culture plates and large bore tubing and thus call it a macrofluidic model to differentiate it from the much smaller microfluidic models.

## Results

### Fabrication of the macrofluidic device

**Figure 1:**
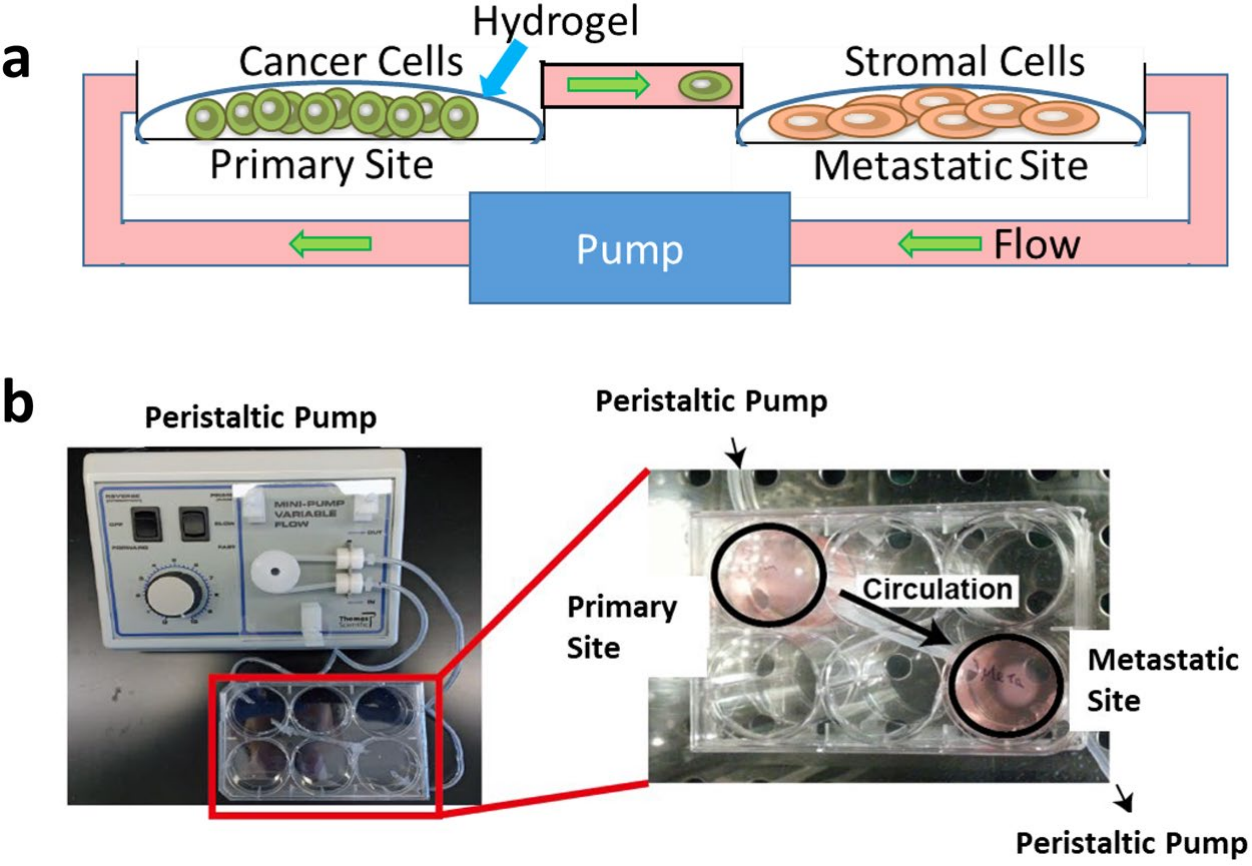
The macrofluidic model. (**a**) Schematic concept of the macrofluidic model. (**b**) Set-up of the macrofluidic model

Our overall plan was to create a closed system recirculating device that would resemble a primary tumor and metastatic sites (Fig. 1a). Cell culture medium reservoirs were made using individual wells of 6-well dishes. One of the wells at one corner and the other well at the far distant corner was used (Fig. 1b). The outlet in the well designated as the “primary site” was connected via a PE tube that was coated with collagen I to the inlet connected to the well designated as the “metastatic site.” Then from the metastatic site outlet a PE tube that was coated with collagen I was connected to a peristaltic pump which controlled the flow to a PE tube connected to the primary site inlet.

### Determining if prostate cancer cells in a hydrogel can migrate in a macrofluidic model

**Figure 2:**
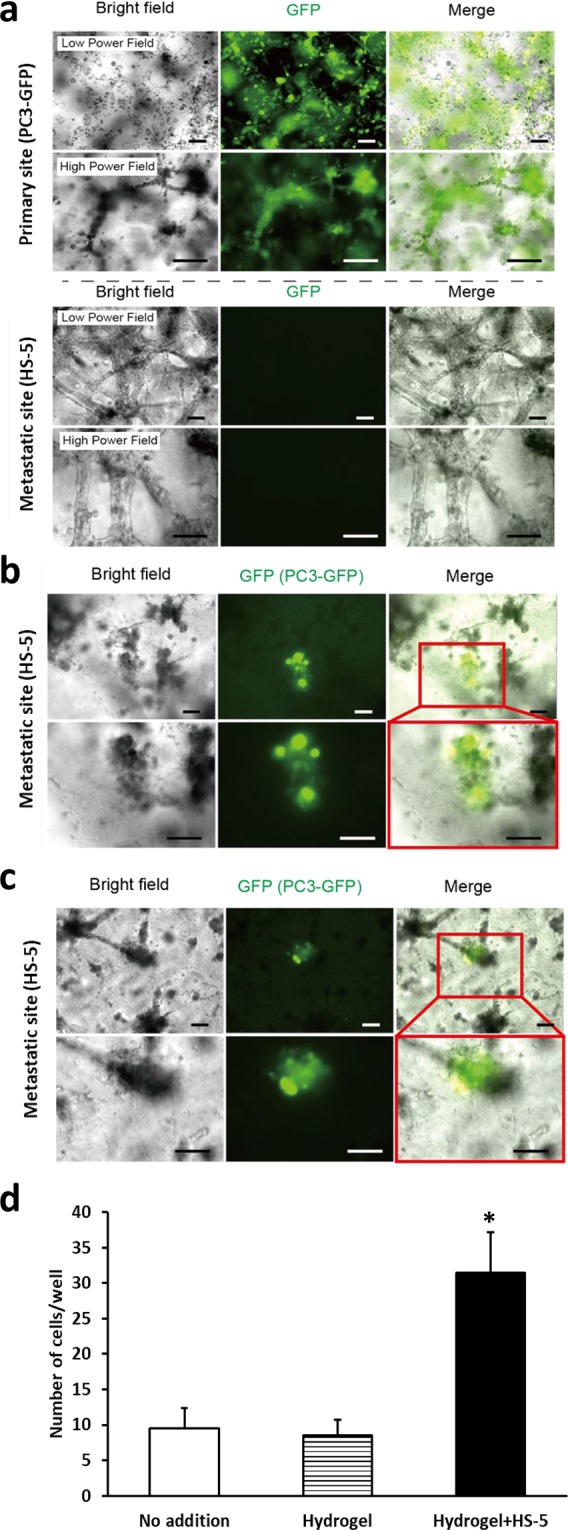
Use of cell lines to create primary tumor in a macrofluidic model. HS-5 cells and green fluorescent protein (GFP) positive PC-3 cells were each mixed in a hydrogel solution and then plated in the metastatic and primary sites, respectively. After the hydrogel solidified the pump was turned on and 48 hours later wells were imaged for cells. (**a**) Bright field and fluorescent microscopic images of primary and metastatic sites at time of establishing macrofluidic model. Note GFP positive (green) PC3-GFP cells in hydrogel in the primary site, but none are visible in the metastatic site (Scale bar: 100 μm). (**b**,**c**) Bright field and fluorescent microscopic images of the metastatic site 48 hours after establishment of macrofluidic model. Note GFP positive PC3-GFP single cells (**b**) or cluster (**c**) in the metastatic site (Scale bar: 100 μm). (D) GFP positive PC-3 cells were mixed in a hydrogel solution and plated in the primary site of the macrofluidic device in combination with either no addition (i.e. culture well surface alone); hydrogel alone or hydrogel plus HS-5 cells at the metastatic site. After the hydrogel solidified the pump was turned on and 48 hours later wells were imaged for cells and the number of cells per well counted. Data are shown as mean ± SD. *P < 0.05 versus no addition and versus hydrogel. N = 3 per group; the experiment was performed twice.

We first wanted to determine if cells incorporated into a hydrogel at one site could migrate to another site in the macrofluidic model. To accomplish this, a volume of 10 μl of PC3-GFP cells (2.0 × 10^6^/ml) or HS-5 cells (2.0 × 10^6^/ml) were mixed with 100 μl of the collagen hydrogel mixture which was then plated into the primary tumor site well and metastatic site well, respectively. After one hour of allowing the hydrogel to set, GFP positive cells were identified in the primary site and we also confirmed that the metastatic site had no GFP positive cells (Fig. 2a). The pump was then turned on and the media flow rate in was maintained at approximately 0.17 ml/min, which has been shown to be the blood flow rate in the rabbit metaphysis^23^. After 48 hours of circulation, the metastatic site was visualized using a fluorescent microscope. Individual GFP positive cells (Fig. 2b) or cell clusters (Fig. 2c) were observed at the metastatic site. To further determine if the presence of HS-5 cells influenced the migration of cells from the primary to the metastatic site, we established macrofluidic models with PC-3-GFP cells in hydrogel at the primary site and either nothing, hydrogel alone or HS-5 cells in hydrogel at the metastatic site. After 48 hours, we counted the PC-3-GFP cells at the metastatic site. PC-3-GFP cells were found in both the culture well alone and the hydrogel alone (Fig. 2d). However, the presence of HS-5 cells in the hydrogel increased the number of PC-3-GFP cells at the metastatic site compared to an absence of HS-5 cells (Fig. 2d). These results indicate that the presence of HS-5 cells at the metastatic site promotes the ability of prostate cancer cells from primary tumor site to localize to the metastatic site.

### Determining if prostate cancer cells in a xenograft can migrate in a macrofluidic model

**Figure 3:**
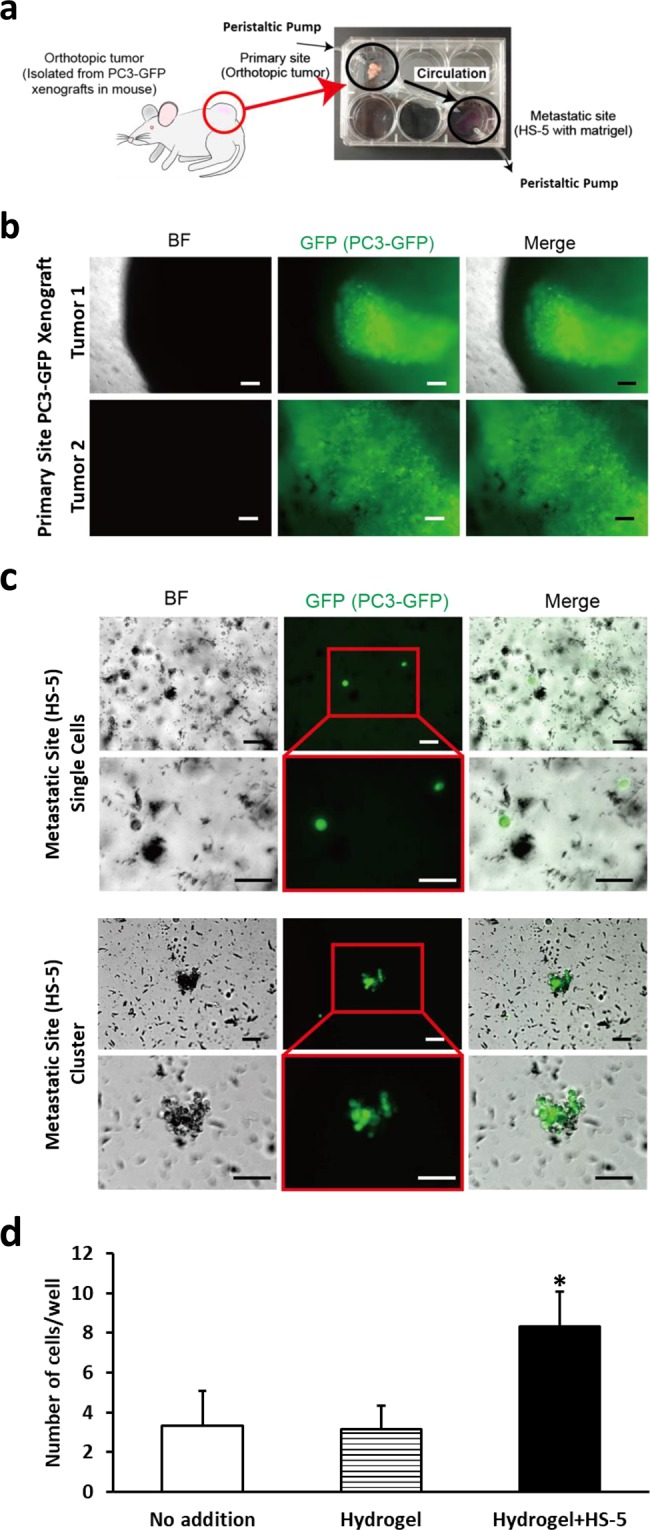
Use of xenograft as primary tumor in a macrofluidic model. A resected PC-3-GFP expressing xenograft and HS-5 cells in a hydrogel solution were plated in the primary and metastatic sites, respectively. After the hydrogel solidified the pump was turned on and 48 hours later wells were imaged for cells. (**a**) Experimental outline where excised xenograft is placed in primary site well. (**b**) Brightfield and fluorescent images of resected PC3-GFP xenograft (GFP, green, Scale bar: 100 μm). (**c**) Brightfield and fluorescent microscopic images of the metastatic site demonstrating GFP positive single cells and cluster (GFP, green, Scale bar: 100 μm). (**d**) A resected PC-3-GFP expressing xenograft was placed in the primary site of the macrofluidic device in combination with either no addition (i.e. culture well surface alone); hydrogel alone or hydrogel plus HS-5 cells at the metastatic site. After the hydrogel solidified the pump was turned on and 48 hours later wells were imaged for cells and the number of cells per well counted. Data are shown as mean ± SD. *P < 0.05 versus no addition and versus hydrogel. N = 3 per group; the experiment was performed twice.

To determine if cancer cells from an intact tumor, including its associated stroma, could migrate to a distant site, we subcutaneously injected mice with the PC3-GFP cells and allowed a tumor to develop to 1 cm3. The xenograft was resected and placed intact into the primary site well (Fig. 3a). A volume of 10 μl of HS-5 cells (2.0 × 10^6^/ml) was mixed with 100 μl of the collagen hydrogel mixture which was then plated into the metastatic site well. The tumor mass containing GFP positive tumor cells was identified in the primary site well (Fig. 3b). Once the metastatic site hydrogel was set, the media flow rate in was maintained at approximately 0.17 ml/min. After 48 hours of circulation, the metastatic site was visualized using a fluorescent microscope. Both GFP positive individual cells (Fig. 3c, upper) and cell clusters (Fig. 3c, lower) were identified in the metastatic site. To further determine if the presence of HS-5 cells influenced the migration of cells from the xenograft in the primary to the metastatic site, we established macrofluidic models with PC-3-GFP xenograft at the primary site and either nothing, hydrogel alone or HS-5 cells in hydrogel at the metastatic site. After 48 hours, we counted the PC-3-GFP cells at the metastatic site. PC-3-GFP cells were found in both the culture well alone and the hydrogel alone (Fig. 3d). However, the presence of HS-5 cells in the hydrogel increased the number of PC-3-GFP cells at the metastatic site compared to an absence of HS-5 cells (Fig. 3d). These results demonstrate that presence of HS-5 cells at the metastatic site promotes the ability of prostate cancer cells from the tumor xenograft to localize to the metastatic site. This observation indicates that the macromodel using an intact tumor recapitulates the ability of tumor cells to migrate from the primary tumor site to the metastatic site.

### Determining if prostate cancer cells in a xenograft at the primary site can target human bone at the metastatic site in a macrofluidic model

**Figure 4:**
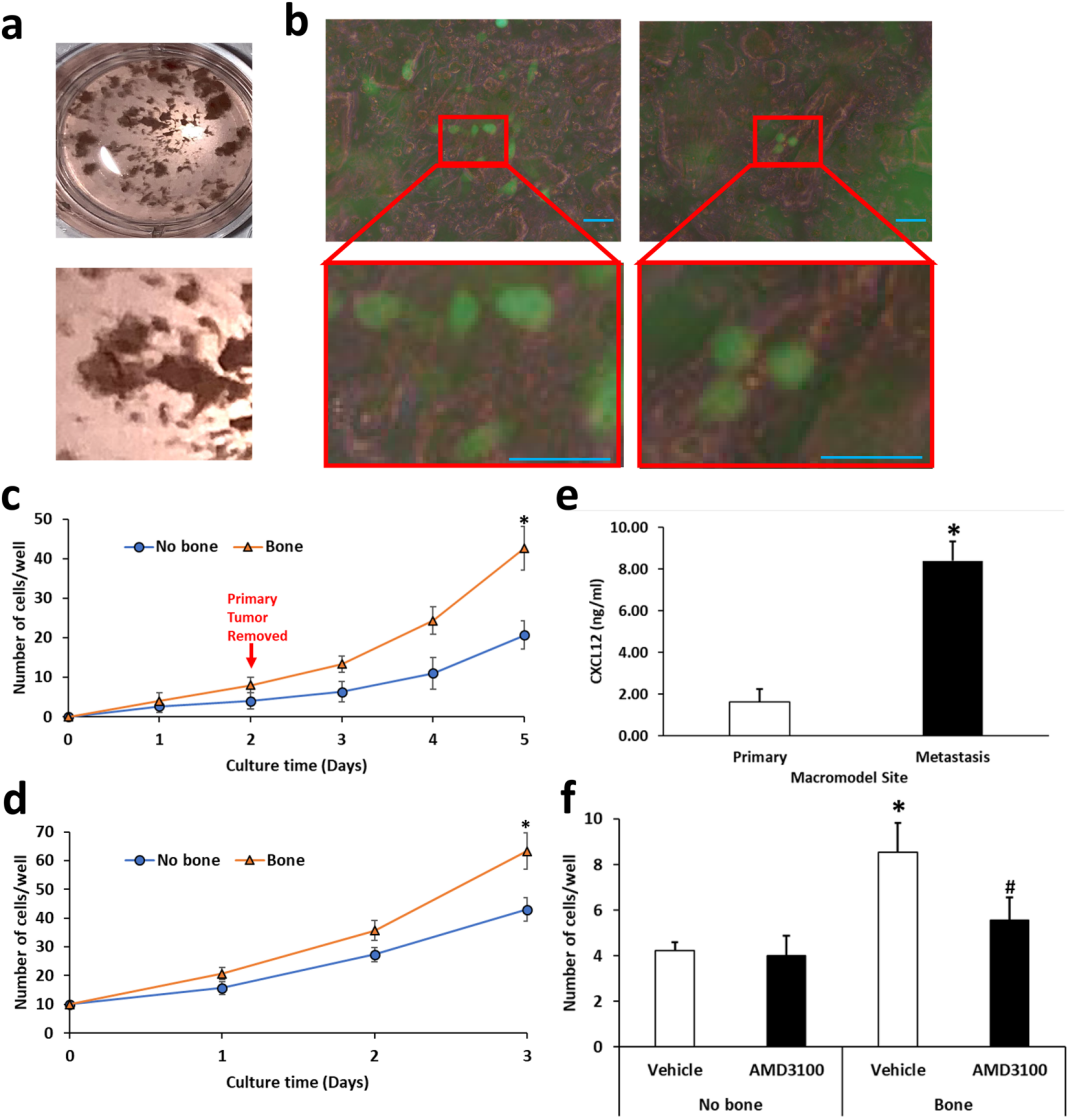
Use of bone chips as metastatic site in a macrofluidic model. C4-2B-GFP xenograft was placed in the primary well and either human bone chips, derived from the femoral head metaphysis, were placed in the metastatic site or no bone chips were placed in a parallel set of models. Multiple units were established to allow collecting on a daily basis. After 48 hours, primary tumor was removed, and cells counted daily for a total culture time of 5 days. (**a**) Bone chips in well. (**b**) Fluorescent images of C4-2B-GFP cells in metastatic site at 48 hours (GFP, green, Scale bar: 50 μm). (**c**) At the indicated days, 2 macromodels each were collected from each group (metastatic site with bone or without bone) and cells counted in the metastatic wells. Data are shown as mean ± SD. *P < 0.05 versus metastatic sites without bone. N = 2 per group; the experiment was performed twice. (**d**) The macromodels were established with nothing in the primary well and a bone chips in the metastatic well. Then 10 C4-2B-GFP cells were plated onto the bone chips and the pump was activated. At the indicated days, 2 models each were collected from each group (metastatic site with bone or without bone) and cells counted in the metastatic wells. Data are shown as mean ± SD. *P < 0.05 versus metastatic sites without bone. N = 2 per group, the experiment was performed twice. (**e**) At day 5, media was collected from primary and metastatic sites from models containing bone and subjected to ELISA for CXCL12. Data are shown as mean ± SD. *P < 0.05 versus primary site. N = 3 per group. (**e**) Models with C4-2B-GFP xenografts in the primary site and with or without bone in the metastatic site were established and some received vehicle or AMD3100 (final concentration 25 μg/ml) and at 48 hours cells in the metastatic site wells were counted. Data are shown as mean ± SD. *P < 0.05 versus metastatic sites without bone. ^#^P < 0.05 versus vehicle in the metastatic site with bone. N = 3 per group; the experiment was performed three times.

We next wanted to determine if the macrofluidic model would function if the metastatic site contained human bone tissue. To perform this, we used the C4-2B cell line which is a bone metastatic cell line created from the LNCaP prostate cancer cell line^24^. We first created a tumor xenograft by subcutaneously injecting mice with C4-2B-GFP cells and allowed the tumors to grow until they reached approximately 1 cm^3^. Tumors were then resected and placed in the primary tumor site well of the macrofluidic device. Bone chips (approximately 0.5 cm^3^) were obtained from trabecular bone from femoral heads that were resected from patients for hip replacement surgery. The chips were placed in the metastatic site of the macrofluidic device (Fig. 4a) and the media flow rate in was maintained at approximately 0.17 ml/min. In parallel, macrofluidic models were established with tumor xenograft in the primary tumor site and in which no tissues were placed in the metastatic site. Multiple macrofluidic model systems were established so that we could collect cells at different time points. GFP positive cells were observed at 24 hours for both the bone chip (Fig. 4b) and non-bone chip metastatic sites; however, by 48 hours there were twice as many GFP-positive cells in the bone chip versus non-bone chip sites (Fig. 4c). To determine if the presence of bone could influence the growth rate in the macrofluidic model, at 48 hours after initiating the cultures, we removed the tumor xenografts from the primary tumor site and maintained the media flow in the macromodel for another 3 days. Each day, through a total of 5 days of culture, a set of macrofluidic models was used to count cell numbers. At the end a total of 5 days of culture, we found that there were more cells and a more rapid growth rate in the metastatic sites with bone chips compared to those without the bone chips even in the absence of primary tumor (Fig. 4c) indicating cell proliferation was occurring. However, it was unclear if this final increase in cell number was due to the presence of more cells in the bone versus non-bone macrofluidic models at the time of primary tumor removal on day 2. To further examine for this possibility, we established macrofluidic models with nothing in the primary site, bone chips in the metastatic site and plated 10 cells per well (10 μl of 1 × 10^3^ cells/ml) on the bone chips in the metastatic site. In order to replicate the growth time of the previous experiment in which we removed the primary tumor and allowed the cells to grow for 3 additional days, we allowed the cells to grow for 3 days after plating them onto the bone chips. At day 3, there were more cells in the metastatic sites with bone versus no bone (Fig. 4c).

An important aspect of tumor metastasis is the presence of circulating tumor cells (CTCs). To determine if the macrofluidic model captured this aspect of metastasis, we evaluated for the presence of PCa cells in the circulating media by collecting media from both the tubing that exited the primary tumor site and the tubing that exited the metastatic site at 48 hours of culture and counting the cells present in the media. We found the presence of C4-2B-GFP cells in the media from both the primary site tubing (4.67 ± 1.53 cells/ml; n = 3, data shown as mean ± SD) and the metastatic site tubing (4.33 ± 1.53 cells/ml; n = 3, data shown as mean ± SD). These results demonstrate the presence of CTC-like cells in the macrofluidic model system.

Taken together, these results demonstrate (1) bone in the metastatic site can be targeted by prostate cancer cells in the primary site in the macrofluidic model and (2) that the presence of bone chips, as opposed to no bone chips, favors prostate cancer cell growth at the metastatic site in the macrofluidic model.

### Chemotaxis in the macrofluidic model

Chemotaxis is an important component of the metastatic cascade^5,25^. In the case of prostate cancer bone metastasis, it has been shown that the bone produces the chemokine CXCL12 (also called stromal-derived factor-1 (SDF-1)), resulting in a gradient of CXCL12 with high levels in the bone compared to the systemic circulation^26,27^. It is well established that prostate cancer cells express the CXCL12 receptor, CXCR4^28,29^ and they respond to the bone-produced CXCL12 gradient by migrating to the bone^30^. To determine if a gradient for CXCL12 was established in the macrofluidic model, at the end of the study on day 5, cell culture conditioned-media collected from the primary tumor site well and the metastatic site well were subjected to ELISA for CXCL12 expression. We found that CXCL12 expression was higher in the metastatic tumor site well compared to the primary site well (Fig. 4e). To confirm that CXCR4:CXCL12 axis contributes to the seeding of the bone chip at the metastatic site, we repeated the tumor xenograft at the primary site and the human bone at the metastatic site experiment with the addition of AMD3100, a CXCR4 inhibitor. In the absence of bone, AMD3100 had no impact compared to vehicle on the number of cancer cells that seeded the metastatic site (Fig. 4f). In contrast, in the presence of bone, AMD3100 partially reduced the number of cells, compared to vehicle, that seeded the bone chips in the metastatic site at 48 hours (Fig. 4e). These results indicate that CXCL12 contributes, in part, to the seeding of the bone chips by the prostate cancer cells in the macrofluidic model. Taken together, these experiments demonstrate that the macrofluidic model using bone chips can establish a functional chemotactic gradient of CXCL12 levels, with the highest levels in the metastatic site.

## Discussion

In the current manuscript, we describe a novel *in vitro* model of metastasis. This model encompasses the ability to incorporate tumor tissue and target organ tissue within multiple compartments attached to a recirculating system. We demonstrated that tumor cells incorporated into a synthesized 3D hydrogel or from an actual excised tumor were able to enter the circulating media as CTC-like cells and mobilize to a distant site. Furthermore, we demonstrated the macromodel allowed for establishment of a functional chemotactic gradient. Thus, this macrofluidic model, while not modeling all aspects of the metastatic cascade, recapitulates several important aspects of the metastatic cascade including chemotaxis, seeding at the target tissue site and cancer cell growth at the metastatic site.

While standard 2D culture systems have enabled great advances towards our understanding of metastasis, they have limitations including atypical cell behavior and suboptimal cell-to-cell communication as the 3D nature of tissues is not represented^31,32^. In order to improve on 2D culture systems, many efforts have been made to create a variety of 3D systems include incorporation of cells into various substrates, such as hydrogels, or hanging drop type systems^33^. Originally these systems did not model aspects of vascular flow, such as shear stress or modulation of biochemical substances that occurs secondary to flow. However, incorporation of microfluidic technologies allowed for recapitulation of vascular flow and provided the opportunity to provide biochemical gradients and physical forces in the culture models^34–37^. In order to optimize modeling of tissues, these systems typically use several different selected cell types to create a synthetic model of tissue either by incorporating the cells into a scaffold or overlaying different cell types on hydrogels. However, these systems only use a small subset of cell types (e.g., tumor cell, osteoblast and endothelial cells^38^) out of the many different cell types that are typically present in clinical tumor tissues (e.g. tumor cell, fibroblast, immune cells, endothelial cells, organ specific cells). Furthermore, they generally will not incorporate the complex non-cellular matrix of the tumor tissue. Thus, while microfluidic systems have provided great advances towards modeling and understanding metastasis, they are limited by only being able to incorporate a limited number of cell types and in ratios that do not match that found in the actual tissues. Furthermore, due to their small size they are, in general, unable to incorporate actual intact normal or diseased tissue and would not capture intratumoral heterogeneity. This latter point is important as intratumoral heterogeneity is a critical aspect of understanding tumor biology and will be missed in the majority of microfluidic models. Thus, the microfluidic models, while having many strengths, have significant limitations. In order to improve on some of the microfluidic model limitations, we created a macrofluidic model that allows for incorporation of actual tissues. Specifically, the macrofluidic model demonstrated that ability to successfully perform *ex vivo* modeling of metastasis using actual intact tumor tissues. These tissues can be derived from patient-derived xenografts (PDX) or from genetically engineered mouse models (GEMM), thus facilitating metastasis studies from both translational and mechanistic perspectives.

This model may be useful for dissecting elements that contribute to metastasis. Specifically, we observed that the number of prostate cancer cells found at the metastatic site differed depending on the cell source at the primary site. Specifically, there were less cells at the metastatic site when the xenograft was placed in the primary site (Fig. 3d) compared to when cells embedded in the hydrogel was located at the primary site (Fig. 2d). There could be several possibilities to account for this observation including that the hydrogel creates a porous structure which could facilitate cell migration into the circulating media compared to the intact tumor that is composed of a basement membrane, extracellular matrix and a variety of cells that interact with the cancer cells through cell adhesion molecules. Another possibility is that the tumor microenvironment found in the xenograft provides a fertile soil including growth factors compared to the hydrogel, thus resulting in less drive for cells to escape from the xenograft. The macrofluidic model will enable future studies to determine the mechanisms that account for these differences.

There are limitations of the macrofluidic model as presented including the lack of a vascular endothelial layer to model intravasation and extravasation. Endothelial cells could be added in future versions of the model system. Another limitation is that it is unclear if the circulating cells derived from the hydrogel or xenograft at the primary tumor site are just passively shed or actively exit the tissue and migrate towards the metastatic site. Furthermore, in the current model, the CTC-like cells do not undergo intravasation or extravasation, thus are not recapitulating that aspect of CTC biology. However, this macrofluidic system could be used to explore this question thorough inclusion of specific chemotactic factors. Finally; whereas, the microfluidic model readily facilitates high throughput screening of therapeutics, the macrofluidic model is not readily amenable to high throughput evaluations. In spite of these limitations, the macrofluidic model has potential to offer some novel aspects to model metastasis including *ex vivo* evaluation of clinically derived tumor tissue, which will incorporate tumor heterogeneity, and the ability to model a complicated metastatic microenvironment including multiple cells types and extracellular matrices.

In summary, we have presented a macrofluidic multi-compartment model system that allows for evaluation of metastasis using both cell suspensions and solid tumors and an *ex vivo* microenvironment. Overall this model is complementary to microfluidic models that have the capability to perform high throughput sophisticated evaluation of cancer cells. The macrofluidic model’s complementary strength is the ability to evaluate relatively large intact tissues of both the primary site and distant target sites. Use of the macrofluidic model will facilitate exploring mechanisms of metastasis.

## Materials and Methods

### Cell lines

The human prostate cancer cell line PC3 was obtained from American Tissue Culture Collection (ATCC) (Manassas, VA). The human prostate cancer cell line C4-2B was a gift from Dr. Leland Chung (Cedars Sinai, Los Angeles, CA). The cells were transduced to overexpress GFP utilizing Lentivirus vector containing GFP gene (PC3-GFP cells and C4-2B-GFP cells). The HS-5 human bone marrow stromal cell line was purchased from ATCC. PC3-GFP and C4-2B-GFP cells were grown in RPMI-1640 (Invitrogen, Thermo-Fisher; Waltham, MA) supplemented with 10% FBS and 1% penicillin/streptomycin (P/S). HS-5 was cultured in DMEM supplemented with 10% FBS and 1% P/S. All cells were serially passaged by trypsinization and maintained at 37 °C and 5% CO2 in a humidified atmosphere. Cell identity was checked every 6 months using short tandem repeat evaluation.

### Tubing placement

To create a circulatory loop, polyethylene (PE)-based Tygon S-50-HL tubing with an inside diameter 1/16 inch and outside diameter 1/8 inch (Saint-Gobain, Inc., France) was used. To maintain consistency with the collagen-I coated cell culture plates and maintain cell viability, the all tubing used in the macrofluidic device was coated with collagen I using a modification of previously described collagen I coating techniques^39,40^. Briefly, the tubing was filled with 100 mg of rat tail collagen I high protein (Sigma; St. Louis, MO) diluted in 100 mls of PBS and incubated at 37 °C for 30 minutes at which time the liquid was removed. To ensure the tubing was coated, we removed and stained a small section of tubing with a Coomassie-brilliant blue solution, which stains the collagen blue. (Sigma) A 1/8 inch drill bit in an electric drill was used to create holes in the sides of the wells of a 6-well cell culture plate at a height of 0.5 cm above the bottom of the well. The tubing was sealed into the holes using Momentive RTV108 One Part Translucent Silicone Sealant and trimmed so that it is flush with the inside of the well. The microfluidic culture system was sterilized by exposing to ultraviolet light in a biosafety cabinet for 60 minutes.

### Hydrogel synthesis

Type I collagen I (8 mg/ml, Corning, Corning, NY) was mixed with the 10x DMEM and 0.8 M NaHCO3 at a 1000 (collagen)/128 (DMEM)/40 (NaHCO3) volume ratio before mixing with each cell line. This mixture was incubated for 60 min inside a humid chamber to form a hydrogel.

### Tumor xenografts

PC3-GFP (2 × 10^6^ cells/mouse) or C4-2B-GFP cells (8 × 10^6^ cells/mouse) were subcutaneously injected into the right flanks of eight-week old male nude mice. When tumors reached 1cc, mice were anesthetized, and tumors resected for use in the microfluidic device. All animal protocols were approved by the University of Michigan Institutional Animal Care and Use Committee. All animal studies were performed in accordance with the relevant guidelines and regulations.

### Bone chips

All human tissue sample procurement was approved by the University of Michigan Medical School Institutional Review Board (IRBMED) and performed with the patients’ informed consent. All methods were performed in accordance with the relevant guidelines and regulations, as required by the IRBMED. Femoral heads were obtained as surgical waste from patients that had undergone hip replacement surgery. Trabecular bone was collected from the femoral head metaphysis manually using a rongeur. Bone chips (approximately 0.5 mm^3^) were placed directly into the metastatic site wells of 6-well plates.

### Measurement of CXCL12 levels

Conditioned media was collected and subjected to CXCL12 ELISA (Human CXCL12/SDF-1 alpha Quantikine ELISA Kit; R&D Systems, Minneapolis, MN) as directed by the manufacturer.

### Cell counts

The metastatic site wells were washed with PBS and trypsinized and rewashed in PBS and resuspended in 100 μl. The cells were then counted using a hemocytometer.

### Inhibition of the CXCR4:CXCL12 axis

AMD3100 (Sigma) was dissolved in PBS and added to the macrofluidic model systems at a final concentration of 25 μg/ml as previously described^41^.

### Statistical analysis

Statistical analysis was performed using GraphPad Prism 5 (GraphPad Software, San Diego, CA) and Microsoft Excel (Microsoft, Redmond, WA). Student’s t-test for single comparions or ANOVA and Fisher’s post hoc test for multiple comparisons were used for analysis and p < 0.05 was considered as statistically significant. All experiments were performed independently two to three times.

## Data Availability

No datasets were generated or analysed during the current study.
